# Cytochrome b5 occurrence in giant and other viruses belonging to the phylum *Nucleocytoviricota*

**DOI:** 10.1038/s44298-025-00091-3

**Published:** 2025-02-11

**Authors:** David C. Lamb, Jared V. Goldstone, Djamal Brahim Belhaouari, Julien Andréani, Ayesha Farooqi, Michael J. Allen, Steven L. Kelly, Bernard La Scola, John J. Stegeman

**Affiliations:** 1 Faculty of Medicine, Health and Life Sciences, Swansea University, Swansea, Wales SA2 8PP UK; 2 Biology Department, Woods Hole Oceanographic Institution, Woods Hole, Massachusetts, 02543 USA; 3 Department of Veterinary Pathobiology, School of Veterinary Medicine & Biomedical Sciences, Texas A&M University, College Station, TX USA; 4 Microbes Evolution Phylogeny and Infection (MEPHI), UR D-258, Aix-Marseille University, Marseille, France; 5 IHU Méditerranée Infection, Timone Hospital, 19-21 Bd Jean Moulin, Marseille, 13005 France; 6 Department of Biosciences, College of Life and Environmental Sciences, University of Exeter, Stocker Road, EX4 4QD Exeter, UK

**Keywords:** Biochemistry, Microbiology

## Abstract

Cytochrome b5 is an electron transport protein found in eukaryotes and bacteria, and plays roles in energy production, lipid biosynthesis and cytochrome P450 biochemistry. Here we report that genes for cytochrome b5 occur broadly among viruses in the class Megaviricetes isolated from the deep ocean, freshwater and terrestrial sources, and human patients. Transcriptional analysis showed that *Mimivirus bradfordmassiliense* cytochrome b5 is expressed in the host and has characteristic spectral properties. Viral cytochrome b5s have either a unique N-terminal transmembrane anchor or are predicted to be soluble proteins. Virus cytochrome b5 proteins share 45–95% sequence identity with one another but no more than 25% identity with that in *Acanthamoeba castellanii*, a host for many giant viruses. Thus, the origin of cytochrome b5 genes in giant viruses remains unknown. Our findings raise questions regarding the evolution and diversity of cytochrome b5, and about the origin of viral haemoproteins in general.

## Introduction

Giant viruses, discovered just over 20 years ago, are enigmatic members of the global microbiome^1^. Traditionally, viruses were defined as biological entities on the edge of life, lacking cellular structure and metabolism, and only reproducing in a host cell. That view of viruses changed in 2003 with the discovery of a giant virus, *Mimivirus bradfordmassiliense* (formerly known as Acanthamoeba polyphaga mimivirus or APMV)^2,3^. (We refer to *Mimivirus bradfordmassiliense* as mimivirus.) The mimivirus genome epitomized an emerging and diverse group of viruses, then referred to as the nucleocytoplasmic large DNA viruses (NCLDV). Giant viruses, many larger than 0.2 μm and with genomes greater than 300 kbp, continue to be discovered in—or captured with—amoebae^4^, and have been identified in a wide range of hosts, including algae^5^. They are increasingly found in metagenomic datasets as well^6,7^. With the growing number of giant viruses, a new classification was developed including a new class, the *Megaviricetes* within a new phylum, the *Nucleocytoviricota*^3^. Especially prominent to-date are members of the *Mimiviridae, w*ith AT-rich DNA genomes, and the proposed *Pandoraviridae* family, which have GC-rich DNA genomes. Multiple clades and species occur in both families. They have been isolated from marine, freshwater, and/or terrestrial sources in different parts of the world^8^.

The first giant virus discovered, *Mimivirus bradfordmassiliense*, encodes more than 1000 proteins, but fewer than 300 have predicted functions^2,9^. Many mimivirus sequences encode proteins not found in any other virus, including protein translation enzymes (thought to be a signature of cellular organisms), DNA repair pathway components and putative homologues of enzymes involved in eukaryotic metabolism^2^. Since publication of the first mimivirus genome, numerous other metabolic genes thought only to be found in cellular lifeforms have been found in diverse GV genomes from different environments. Using bioinformatic approaches, we and others have identified GV genes and proteins and their orthologues that are involved in known cellular primary metabolic pathways *e.g*. glycolysis, gluconeogenesis, TCA cycle, photosynthesis and lipid β-oxidation^10–12^. It is already accepted that viruses can hijack host metabolic networks, but the GVs auxiliary metabolic genes represent another form of host metabolism manipulation, by expanding, rather than merely enhancing, host cells’ catalytic capabilities especially in harsh variable environments. However, the functionality of these genes remains unclear, and how they integrate into established host metabolic systems is completely unknown^13^.

**Fig. 1:**
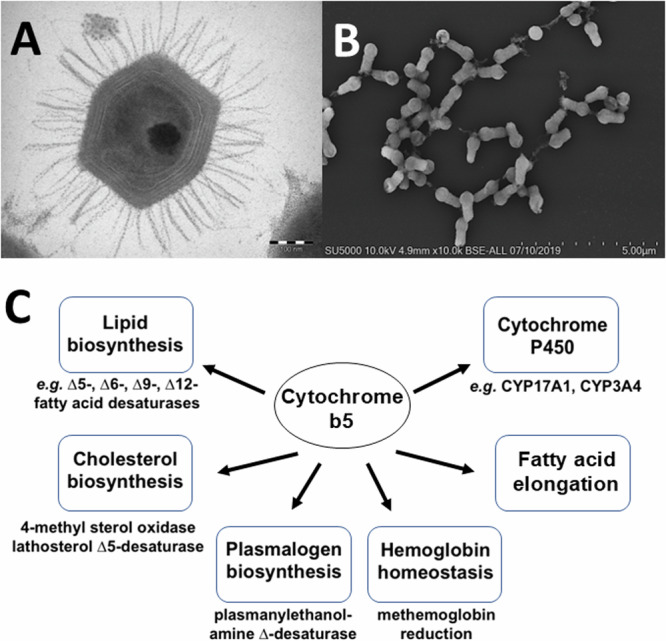
Prominent viruses and cytochrome b5 roles. **A** Transmission electron microscopy image of a *Mimivirus bradfordmassiliense* particle. The sample was embedded in resin, ultrasectioned, and imaged using either a Morgagni 268D transmission electron microscope. **B** Scanning electron microscopy image of *Tupanvirus salinum* viral particles. Particles were fixed, centrifuged onto slides, metalized, and imaged using an SU5000 SEM (Hitachi, Japan). **C** Examples of mammalian cytochrome b5 redox partners and roles in biochemical pathways.

Heme proteins are among the most versatile of metalloproteins found in nature and play essential roles in diverse and distinct biological functions from gas exchange to redox reductions^14^. Cytochrome b5, originally detected in the larvae of the silkworm *Platysumia cecropia* by Sanborn and Williams in 1950^15^, is a ubiquitous electron transport protein found in animals, plants, fungi and purple photosynthetic bacteria^16^. Soluble forms of cytochrome b5 function in photosynthetic energy production in bacteria and the reduction of hemoglobin in animals^16,17^. In contrast, membrane-bound forms of cytochrome b5 function in the mitochondria and the endoplasmic reticulum (ER) of eukaryotes. ER-associated cytochrome b5 roles are shown in Fig. 1 and include roles in fatty acid biosynthesis driving fatty acid desaturase enzymes (*Δ*9-, *Δ*6-, *Δ*5- and *Δ*12-desaturases), in cholesterol biosynthesis, and in the reduction of cytochromes P450 enzymes where cytochrome b5 has been shown to alter substrate metabolism by different P450s^16^. No function has been attributed to cytochrome b5 in the mitochondria^18^.

Previously a gene encoding a putative cytochrome b5 protein was found in the large double-stranded DNA virus *Ostreococcus tauri* virus OtV-2^19^, and the protein was expressed and characterized. This soluble viral cytochrome b5 protein had near identical spectral properties to purified membrane-bound, recombinant human cytochrome b5^19^. The crystal structure of the *O. tauri* OtV-2 cytochrome b5 revealed a single domain, comprising four β sheets, four α helices and a heme moiety, which is similar to that found in larger eukaryotic cytochrome b5 proteins^19^. The *O. tauri* OtV-2 cytochrome b5 also was shown to reduce a eukaryotic cytochrome P450 (*Candida albicans* CYP51) through the transfer of reducing equivalents, suggesting this viral cytochrome b5 can act as a bona fide cytochrome b5 in P450 reactions, as well as potentially be involved in modulating lipid and sterol biosynthetic enzyme activity. This added credence to our current hypothesis that novel viral heme proteins, including viral cytochrome P450 enzymes^20^, can modulate either viral or host sterol or lipid content to maximize energy production and facilitate viral replication. This possibility has particular relevance to viruses that have lipid envelopes and/or rely on controlled passage through the membrane for virion release.

The current work describes the discovery of multiple cytochrome b5 enzymes in numerous giant virus genomes and from metagenome datasets that are currently publicly available. Identification of enzyme systems that seemingly cannot function in the inert virion particle yet are identifiable across a wide range of viral species and ecotypes, points to key aspects of NCLDV biology that remain unexplored.

## Results

### Overall distribution of cytochrome b5 in the virosphere

**Fig. 2:**
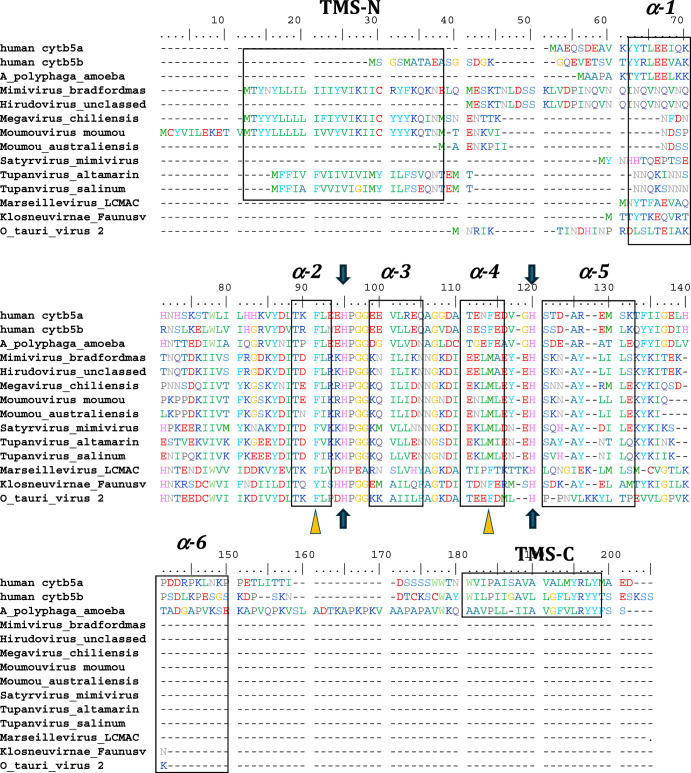
Amino acid sequence alignment of viral cytochromes b5. Alignment of human cytb5a (endoplasmic reticulum), human cytb5b (mitochondrial), amoeba (*A. castellanii*) cytb5, and cytb5 of selected viruses including, the previously reported *Ostreococcus tauri virus 2* cytb5. The schematic arrangement of secondary structures of the proteins obtained from cytb5 structures (PDB ID: 4HIL, 1AW3) is shown above the alignment. N- and C- terminal transmembrane regions are indicated as TMS-N and TMS-C. Blue arrows denote the heme-bound histidine residues, and yellow triangles show important conserved heme-adjacent F40 and F63 (or M, see text). See also Fig. 8. Negatively charged residues at positions 94–95, 99–100, 109, and 116 have been previously shown to interact with cytochrome P450 enzymes. See Supplemental Table 2 for accession numbers and full species names where available.

Among the viruses, we identified numerous cytochrome b5 genes encoded in the genomes of specific groups within the *Nucleocytoviricota* including *Ostreococcus* viruses, which are atypical giant viruses, based on the absence of RNAPol2. Genes were assigned as encoding cytochrome b5 based on the conservation of sequence and secondary structure identity with known eukaryotic cytochromes b5. Key factors in sequence assignment include the retention of secondary structure elements occurring in the order β1-α1-β4-β3-α2-α3-β5-α4-α5-β2-α6, the absolute conservation of two histidine residues residing in the loops of between helices α2 and α3 and between helices α4 and α5, and the overall domain identification using Pfam models (Fig. 2). The conservation of the two α2-α3 and α4-α5 histidine residues is key, as these conserved histidine imidazoyl side chains bind the heme cofactor in authentic cytochrome b5 proteins.

**Fig. 3:**
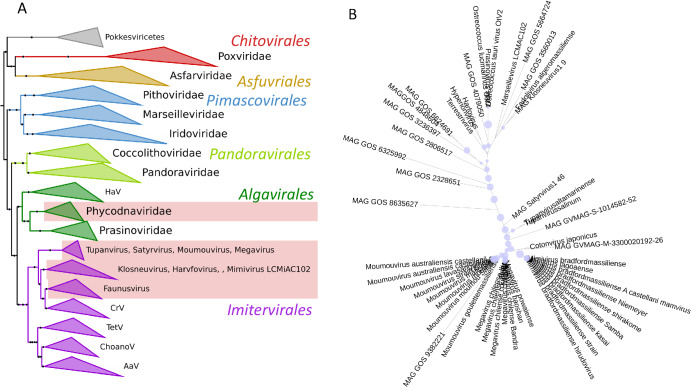
Phylogenetic analyses of *Nucleocytoviricota* cytochrome b5 proteins. **A** *Nucleocytoviricota* phylogeny based on marker genes showing clades with cytochrome b5.The 7-marker gene *Nucleocytoviricota* phylogeny was modified and pruned from Aylward (3), retaining their nomenclature and marker genes for guidance. The pale red blocks indicate the presence of cytochrome b5 in these clades. AaV *Aureococcus anophagefferens* virus; ChoanoV choanoflagellate virus, CrV *Cafeteria robergensis* virus, HaV *Heterosigman akashiwo* virus, TetV tetraselmis virus. **B** Phylogeny of viral cytochrome b5 proteins. The maximum likelihood phylogenetic tree shows the relationship of the viral cytochrome b5 proteins. Bootstrap values are represented as proportional blue circles at nodes, with the largest blue circle corresponding to a bootstrap value of 100%. Figures prepared using iTol.

A phylogeny of the *Nucleocytoviricota* with the branches that contain cytochrome b5 genes highlighted, appears in Fig. 3A, and details of the viral cytochromes b5 are presented in Table 1. Thus far, our searches revealed cytochrome b5 genes to be present in the *Mimiviridae* genera *Mimivirus*, *Moumouvirus* and *Megavirus* (formerly known as Groups A, B and C respectively) and in tupanviruses (*Tupanvirus altamarinense and Tupanvirus salinum)*. Cytochrome b5 genes were also detected in giant virus genomes assembled during the analysis of metagenomic data from terrestrial soils (satyrvirus, harvfovirus, hyperionvirus and terrestrivirus) and from marine sediments (marseillevirus and pithovirus)^21^. In addition, cytochrome b5 genes were retrieved from the genomes of NCLDV that infect the green algae *Ostreococcus lucimarinus and O. tauri* (the Phycodnavirus OtV-2 mentioned above)^22^.

**Table 1:** Cytochrome b5 features

Virus	Location	Total ORFs	Protein length	Trans-membrane region	Genbank accession number
Mimivirus	U.K.	1262	124	5-22	Q5UR80
Mamavirus	France	988	118	6-22	AEQ60829
Oyster virus	Brazil	969	89	-	AKI79407
Kroon virus	Brazil	930	86	-	AKI80364
Hirudovirus	Tunisia	930	95	-	AHA45216
Niemeyer virus	Brazil	1003	124	6-23	ALR84216
Bombay virus	India	898	124	6-23	AMZ03071
Shirakomae virus	Japan	986	95	-	BAV62730
Kasaii virus	Japan	988	95	-	BAV61744
Samba virus	Brazil	971	95	-	AHJ40257
Moumouvirus	France	894	111	12-33	AGC02191
*Moumouvirus monve*	France	1150	111	12-33	AEX62481
Saudi moumouvirus	S-Arabia	953	111	12-33	AQN68565
*Moumouvirus goulette*	France	970	112	12-33	AGF85059
*Moumouv. australiensis*	Australia	903	74	-	AVL95051
*Moumouvirus maliensis*	Mali	845	112	12-33	QGR54182
Borely moumouvirus	Brazil	934	112	12-33	QID06377
*Megavirus chiliensis*	Chile	1120	103	6-22	AEQ33147
Mimivirus lba	France	1176	77	-	AGD92703
Megavirus courdo11	France	1337	77	-	AFX92855
Powai Lake Megavirus	India	996	103	6-22	ANB50850
Bandra Megavirus	India	1055	103	6-22	AUV58705
*Megavirus vitis*	France	1027	77	-	AVL94070
Mimivirus sp SH	China	947	103	6-22	AZL89108
*Tupanvirus altamarinense*	Brazil	1276	98	1-18	QKU33443
*Tupanvirus salinum*	Brazil	1359	98	1-22	QKU34676
Satyrvirus sp.	USA	906	76	-	AYV84960
Marseillevirus LCMAC102	marine sediment	456	78	-	QBK86278
*O. lucimarinus* virus					
Olv1	France	250	91	-	ADQ91589
Olv2	USA	269	92	-	YP_009172721
Olv3	Chile	248	92	-	AFK66042
Olv4	Canada	299	91	-	AET84663
Olv5	France	251	91	-	AGH31115
Olv6	France	248	91	-	AFK65792
Olv7	USA	243	91	-	ALI95841
*O. tauri* virus					
OtV2	Ocean	237	91	-	CB170200
*Homo sapiens*		21000	134	113-129	NP_683725
		15455	141	119-137	ELR24118

In bacteria and eukaryotes there are multi-domain proteins that include a cytochrome b5 domain. A phylogenetic tree of protein families that include in their structure a cytochrome b5-domain-containing motif is presented in supplemental information (Supplemental Fig. 1), showing where viral cytochrome b5 proteins fit among the multi-domain proteins. Although some metagenomes have genes coding for multidomain proteins that include cytochrome b5 domains, the cytochrome b5 proteins described here are single domain proteins. Figure 3B shows a phylogenetic tree of cytochrome b5 proteins.

### *Mimiviridae* cytochrome b5 genes

Searching the first giant virus genome sequence^2^, *Mimivirus bradfordmassiliense* (Fig. 1A), revealed the presence of a gene (MIMI_L628) coding for a 124 amino acid protein containing the canonical eukaryotic cytochrome b5 heme domain (located between residues 49–124). Following this initial observation, we discovered putative cytochrome b5 proteins encoded in giant viruses in all three *Mimiviridae* genera, *Mimivirus, Moumouvirus, and Megavirus* (Table 1). Analysis of the viral cytochrome b5 sequences revealed the putative proteins to be either soluble in nature, existing as a single hydrophilic domain protein, or as presumed membrane bound proteins possessing a single N-terminal transmembrane anchor. This was true both within and between *Mimiviridae* sublineages. For example, in mimiviruses, the *M. bradfordmassiliense* cytochrome b5 (accession number Q5UR80) consists of a hydrophilic heme-bound globular domain with a predicted 16–18 amino acid N-terminal transmembrane anchor, while the hirudovirus cytochrome b5 gene encodes a smaller b5 domain without an encoded recognizable membrane anchor (Table 1; Fig. 2). Similar examples of membrane bound and soluble cytochromes b5 are observed in genera *Megavirus, and Moumouvirus* (Table 1; Fig. 3). The occurrence of an N-terminal membrane anchor is unique to viral cytochrome b5 proteins alone; all eukaryotic cytochrome b5 proteins examined to date have a C-terminal membrane anchor, *e.g*. human and viral amoeba host *A. castellanii* cytochromes b5 (Fig. 2).

There is a strikingly high degree of sequence identity (98–100%) between inferred cytochrome b5 protein homologues in the mimiviruses from different parts of the world. Similarly, there is >95% sequence identity among the cytochrome b5 proteins in *Megavirus* species, and among the cytochrome b5 proteins in *Moumouvirus* species, from different parts of the world. Between groups there is lower conserved sequence identity, with the mimiviruses cytochrome b5s having <60% identity with megavirus or moumouvirus cytochrome b5s. Moumouvirus and megavirus cytochrome b5s share a somewhat higher % identity ( ~ 75%). All of the *Mimiviridae* cytochrome b5 genes are AT-rich (69–75%), like the *Mimiviridae* genomes overall, and nucleotide sequences also are highly similar within subgroups.

**Fig. 4:**
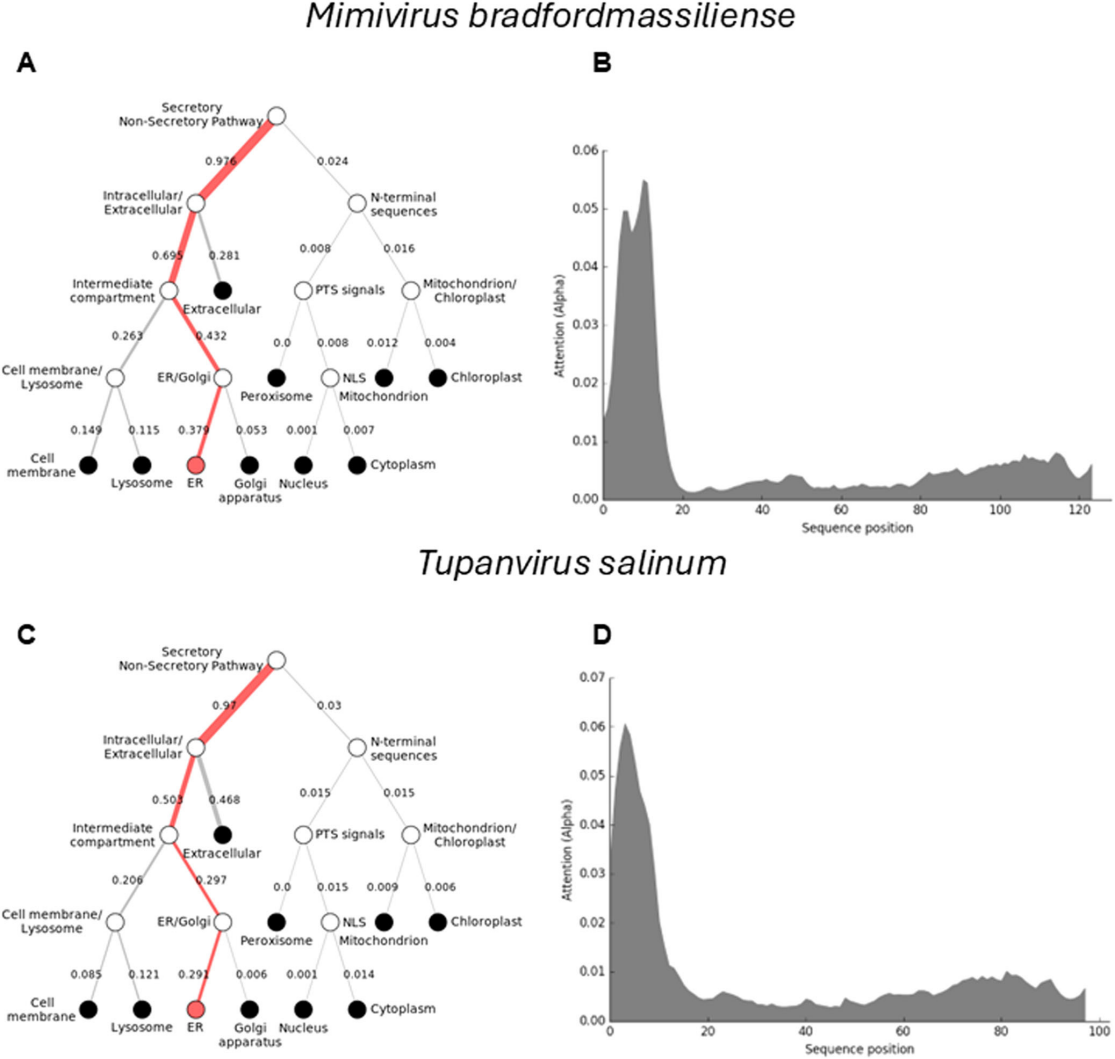
Viral cytochrome b5 subcellular localization predictions. The DeepLoc prediction server indicates that the full-length cytochrome b5 proteins of **(A)** *Mimivirus bradfordmassiliense* and **(C)** *Tupanvirus salinum* are highly likely to be localized to the endoplasmic reticulum (ER), with their signal peptides located in their N-terminal sequences **(B, D)**.

To further understand how the *Mimiviridae* cytochrome b5 proteins utilize their N-terminal transmembrane anchor to interact and colocalize with cellular organelles, we performed a protein colocalization prediction. The *Mimivirus bradfordmassiliense* cytochrome b5 (Q5UR80) is predicted with high likelihood to colocalize with the ER through its N-terminal sequence (Fig. 4). Thus, in the case of mimivirus cytochrome b5 (Q5UR80) the likelihood for the endoplasmic reticulum was 0.8316, indicating an 83% probability of localization, while the likelihoods for other compartments were lower. (See Supplemental Table 2).

However, this was not the case for cytochrome b5s in all the *Megaviricetes*. Some of these cytochrome b5s, such those as in hirudovirus, Oyster virus, and Samba virus, were predicted to be in the cytoplasm, without association with any specific cellular organelle (Supplemental Table 2). This discrepancy could arise from the absence of a discernible membrane anchor in these proteins. Similar observations are noted for viruses in the *Moumouvirus* or *Megavirus* genera (Supplemental Table 2). This finding suggests that viral cytochrome b5 proteins strategically utilize their N-terminal membrane anchor to associate with the ER, potentially implicating these proteins in critical ER-related processes such as lipid and protein synthesis, which are vital for viral replication and assembly.

### Tupanvirus cytochromes b5 genes

The tupanviruses (Fig. 1B), related to *Mimiviridae*, were discovered in a soda lake in Brazil (*Tupanvirus salinum*) and in the deep ocean (3,000-m depth) (*Tupanvirus altamarinense*) and are among the first viruses reported to possess genes for amino-acyl tRNA synthetases for all 20 standard amino acids^23^. Both tupanviruses possess genes predicted to encode cytochrome b5 proteins, each containing a N-terminal transmembrane anchor (Supplemental Table 2; Fig. 4). While the tupanvirus cytochromes b5 share 78% percent amino acid identity, they share between 55–60% identity with cytochrome b5 proteins in the mimiviruses and megaviruses but only 45% identity with moumouvirus cytochrome b5 proteins. Subcellular colocalization predictions for both tupanviruses’ cytochrome b5 proteins showed a strong likelihood of association with the ER, possibly due to an ER-targeted sequence within their N-terminal region (Fig. 4). It is important to note, however, that these results represent predictions generated by algorithms and require experimental validation for confirmation. This observation nevertheless suggests that as with mimivirus, the tupanvirus cytochrome b5 proteins are likely to colocalize with the ER, suggesting its involvement in modulating ER function and potentially exerting influence over various metabolic pathways to enhance viral fitness.

Subcellular localization predictions for the cytochrome b5 proteins of *O. lucimarinus* virus, marseillevirus, and satyrvirus revealed a different pattern. These proteins did not show significant colocalization with the ER. Instead, our prediction results indicated their localization in the cytoplasm, with a weak likelihood of association with specific organelles (Supplemental Table 1). This discrepancy suggests that the subcellular localization of cytochrome b5 proteins in infected hosts vary among different viruses. One possible explanation for this difference could be the absence of a membrane anchor on these proteins, as indicated by our alignment result (Fig. 2). The lack of a membrane anchor could be expected to prevent their association with the ER and other organelles, leading to their predominant localization in the cytoplasm. These findings underscore the complexity of this protein localization, and the diverse strategies employed by viruses to interact with cellular machinery.

### Gene synteny, transcriptome analysis and cytochrome b5 spectral analysis

**Fig. 5:**
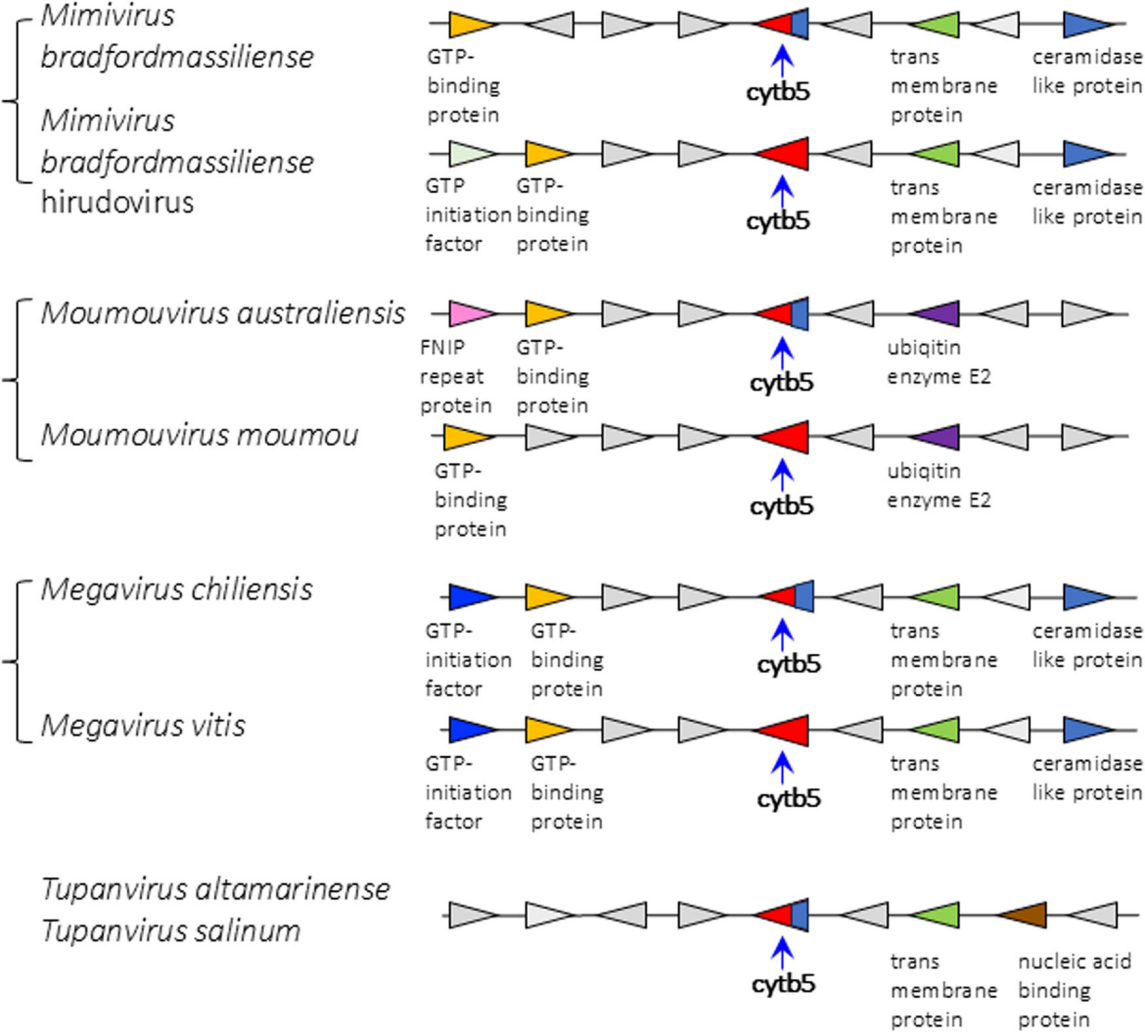
Synteny analysis of cytochrome b5 loci in *Mimiviridae* and tupanviruses. Comparative examples of the loci and surrounding genes for membrane-bound and soluble cytochrome b5s in *Mimiviridae* species, and in tupanviruses. Blue base on the b5 symbol indicates presence of membrane anchor on that sequence.

Viruses in the *Mimiviridae* genera *Mimivirus, Moumouvirus*, and *Megavirus* exhibit high degrees of shared gene order (synteny) extending over 8 genes around the cytochrome b5 gene (Fig. 5). There is less shared synteny of these with tupanviruses (Fig. 5), and no evident sharing of synteny between the *Mimiviridae* and the *Ostreococcus* viruses (Supplemental Fig. 2). It is noteworthy that there is a prevalence of Fe(II)-2-oxoglutarate dependent dioxygenases among the genes flanking cytochrome b5 in the *Ostreococcus viruses*.

**Fig. 6:**
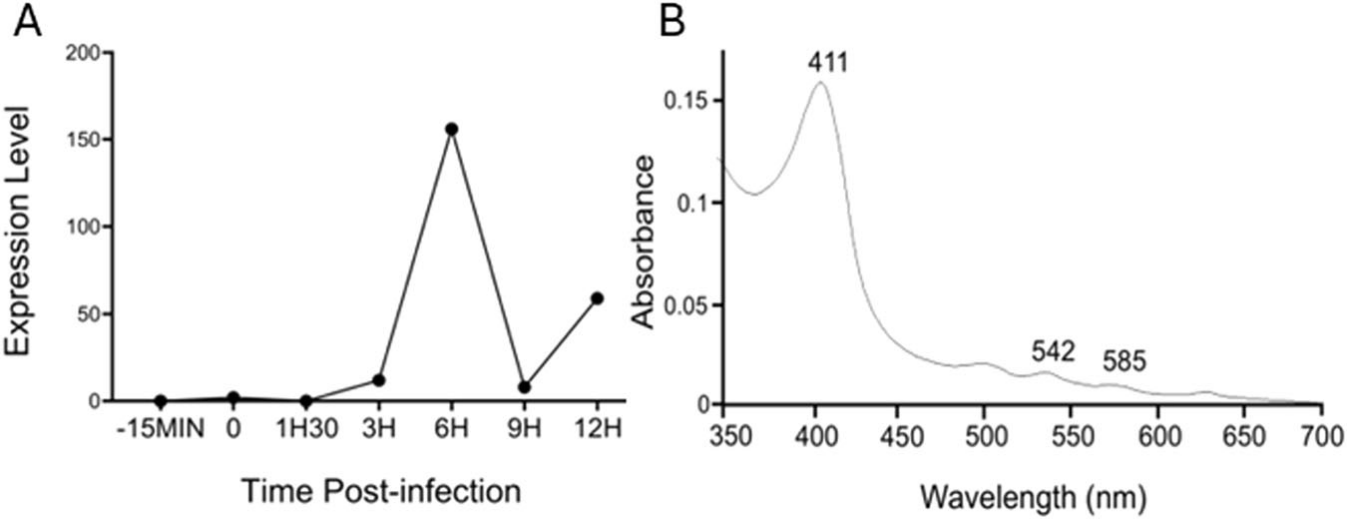
*Mimivirus bradfordmassiliense* cytochrome b5 transcriptomic profile and protein spectrum characterization. **A** Cytochrome b5 expression levels during the virus replication cycle are presented with normalized read counts. **B** Oxidized absorbance spectrum of purified recombinant *M. bradfordmassiliense* L628 (cytochrome b5).

Transcriptomic analysis also revealed that, of the 90% of all mimivirus genes transcribed during infection, the mimivirus gene L628 (which putatively encodes cytochrome b5) is maximally transcribed at 6 h (Fig. 6A)^24^. This expression pattern falls into the ‘late’ class of expression upon mimivirus infection, as do other genes encoding oxidoreductase enzymes, or structural components of the mimivirus particles^24^. Heterologous expression of histidine-tagged mimivirus cytochrome b5 (MIMI_L628) in *E. coli* and purification using nickel affinity chromatography yielded a distinctive hemoprotein. Cell fractionation revealed MIMI L628 to be a membrane bound protein located in the membrane fraction following ultracentrifugation at 100 000 × *g* and solubilised by the use of detergents (1% (v/v) Tween 20 and 1.5% (w/v) sodium cholate). The absorbance spectra of oxidized recombinant MIMI L628 protein (Fig. 6B) shows maxima at 412, 528, and 567 nm. These spectral characteristics are typical of purified cytochrome b5s from eukaryotic organisms described in the literature (18).

### Analysis of cytochrome b5 secondary structure and overall topology

**Fig. 7:**
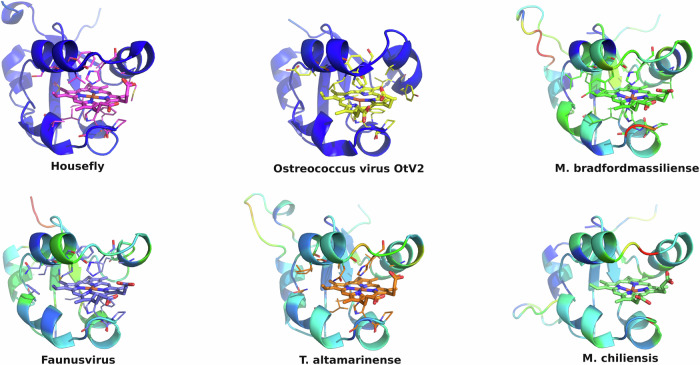
Crystal structure of housefly cytochrome b5 compared with homology models of virus cytochrome b5s. The homology model helices are colored by b-factor (blue = highest confidence, red = least confidence). Viruses are: *Ostreococcus* virus, *Ostreococcus lucimarinus*; Mimivirus, *Mimivirus bradfordmassiliense;* Faunusvirus, from soil metagenome Genbank Accession number AYV79526.1; Tupanvirus, *Tupanvirus altamarinense*; Megavirus, *Megavirus chiliensis*

**Fig. 8:**
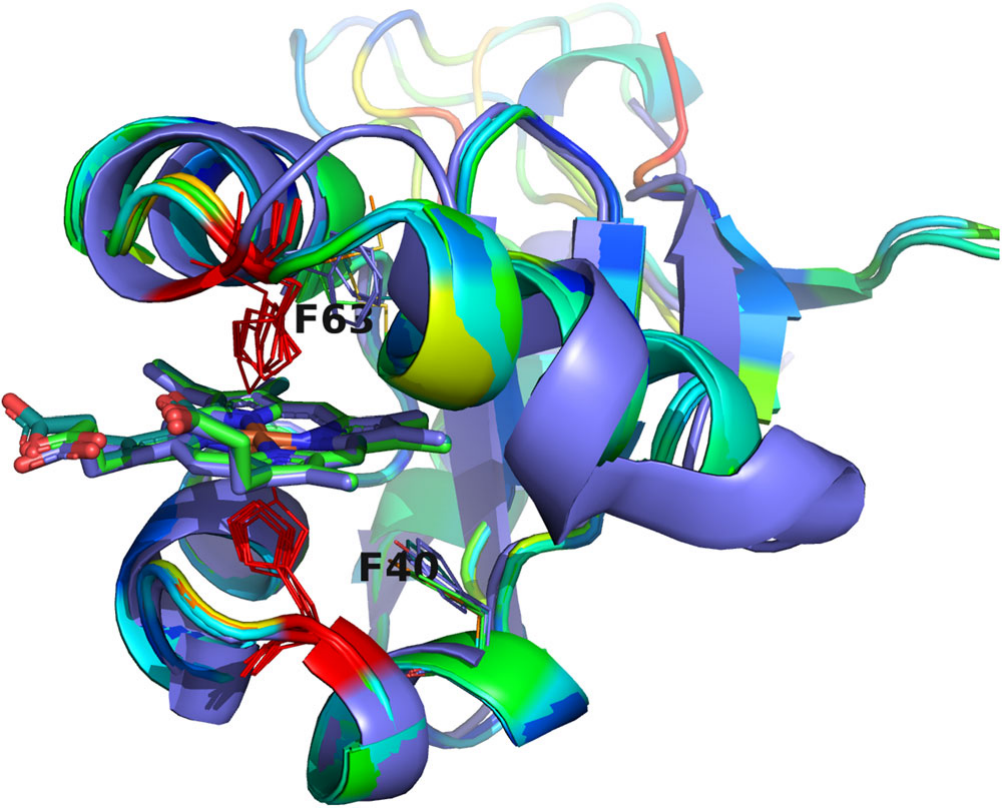
Overlaid viral cytochrome b5 structures showing the positioning of the conserved histidine residues (red) binding the heme. Note that there is some variability in the heme-adjacent residues. Human, amoeba, and virus conserved F40 (alignment position 91) is a tyrosine in faunusvirus, while F63 (alignment position 114) is a conserved methionine in mimivirus, megavirus, tupanvirus, and moumouvirus, but a phenylalanine in soil faunusvirus, hyperion virus, terrestrivirus and metazoan cytochrome b5s.

All eukaryotic ER-bound microsomal cytochrome b5 proteins examined to-date are anchored to the ER lipid bilayer by a C-terminal transmembrane anchor. Mitochondrial cytochrome b5 proteins are also anchored in the membrane by a C-terminal transmembrane anchor. In the case of human and *A. castellanii* microsomal cytochrome b5s, each possesses a single 16–18 amino acid C-terminal region that anchors the protein to the ER membrane (Fig. 2 and Table 1). In contrast, analysis of the viral cytochrome b5 sequences reveals the putative proteins to be either soluble, existing as a single hydrophilic domain protein, or membrane bound apparently through a putative N-terminal transmembrane anchor sequence rather than a C-terminal anchor. Protein modelling demonstrates the high degree of tertiary structural homology between the crystalized house fly and *Ostreococcus* cytochrome b5 and the modelled mimivirus, megavirus, tupanvirus, and faunusvirus cytochrome b5s (Fig. 7). Notably, the heme-binding histidines are spatially conserved (Fig. 8), but there is some variability in the heme-adjacent residues: conserved *Ostreococcus* Phe44 is a tyrosine in faunusvirus; and Phe67 is a conserved methionine in mimiviruses, megaviruses, tupanviruses, and moumouviruses, but a phenylalanine in soil faunusvirus, hyperionvirus, and terrestrivirus cytochrome b5s.

## Discussion

Our search of more than ten thousand completely sequenced and annotated viral genomes revealed cytochrome b5 genes in several virus taxa, including the AT-rich genomes in the *Mimiviridae*, tupanviruses, and *Marseilleviridae*, members of the class *Megaviricetes*, large double stranded DNA viruses. Additionally, we found cytochrome b5 genes in viruses infecting *O. lucimarinus*, similar to the *virus infecting O. tauri* (Table 1). The *Ostreococcus* viruses are also members of the *Nucleocytoviricota*, albeit much smaller than the giant mimiviruses, and lacking RNAPol2 genes. We assigned genes as encoding cytochrome b5 based on the presence of the conserved heme-binding consensus motif found in all cytochrome b5s described to date^16^. The *Mimiviridae* cytochrome b5 genes are not homologous with the *Ostreococcus* virus genes and appear to have been acquired separately, potentially an example of convergent evolution. While genome size is a clear and defining feature for giant viruses, it is interesting to note that OtV2 infects one of the world’s smallest free-living eukaryotes, suggesting an important metabolic role(s) for cytochrome b5 in overcoming the extremes of scale in both the virus and host.

The earlier expression of a recombinant cytochrome b5 from the OtV2 virus, and analysis of activity, showed that at least this virus cytochrome b5 gene encodes a functional protein^19^. Whether cytochrome b5 genes in the other *Ostreococcus* phage and the giant viruses also encode functional proteins is not yet known, although based on structural and transcriptional features the virus cytochrome b5 proteins are almost certainly functional. Larger questions regarding functional role(s) these enzymes play in the viruses, or hosts, and the origin of the virus cytochrome b5 genes are unanswered.

Increasingly, metabolic enzymes encoded by genes in the giant viruses have been found to be functional in vitro and to be expressed in vivo upon infection of a host (10,11). These include genes encoding enzymes involved in glycolysis, gluconeogenesis, tricarboxylic acid cycle, photosynthesis, and β-oxidation. One of the important functions of cytochrome b5 is providing reducing equivalents during the catalytic cycle of cytochrome P450 proteins. This could be considered as a possible function in the *Mimiviridae* and the tupanviruses, where there are multiple P450 genes, and those genes are also expressed in the infected host, shown at least for mimivirus^10^. However, the *Marseilleviridae*, which do have cytochrome b5 genes, do not appear to have genes that encode any cytochrome P450. Moreover, our search revealed cytochrome b5 genes only in the AT-rich *Mimiviridae*, *Marseilleviridae* and the tupanviruses but not in the other branches of the *Megaviricetes* including in the GC-rich pandoraviruses, mollivirus, kaumoebavirus or the orpheovirus, all of which contain cytochrome P450 genes. This would suggest that if cytochrome b5 does support virus P450 function, that so far it would seem to be restricted to AT-rich virus clades.

In addition to its role in in providing electrons to support the mono-oxygenation by cytochrome P450, cytochrome b5 plays pivotal roles in cellular metabolism, particularly in fatty acid elongation, desaturation, and cholesterol biosynthesis^25^. These metabolic pathways are essential for maintaining cellular homeostasis and protecting against metabolic disorders^26^. In the context of viral infection, the presence of cytochrome b5 in giant viruses like mimiviruses and tupaniviruses suggests its potential involvement in similar metabolic processes. Our in silico predictions of subcellular colocalization indicate cytochrome b5’s likely localization within the endoplasmic reticulum (ER), consistent with its predicted transmembrane domain, and suggests that cytochrome b5 expression in the ER may be related to the ER involvement in lipid metabolism. The ER serves as a hub for lipid metabolism, housing enzymes crucial for triglyceride and cholesterol biosynthesis as well as ergosterol biosynthesis in amoebae^27^. During the replication cycles of viruses like the mimiviruses, there is a notable close association between membranes derived from the endoplasmic reticulum (ER) and sites where viral assembly occurs^28^. This suggests that the ER is effectively “hijacked” for lipid synthesis, a process crucial for the formation of virus particle envelopes as shown in some DNA viruses^29^. Importantly, no viral cytochrome b5 reductase has yet been discovered indicating that the viral cytochrome b5s described herein would function in tandem with host cytochrome 5 reductase. Viral cytochrome b5 may serve a function within the ER, participating in the desaturation and elongation of fatty acids. Such a role would ensure the availability of an adequate lipid supply, which is essential for the overall fitness and successful replication of the virus. Moreover, in this case, we speculate that cytochrome b5, by transferring electrons to host enzymes, may facilitate the synthesis of polyunsaturated fatty acids by transferring electrons to both host and virally encoded enzymes involved in lipid metabolism. We note a slight caution regarding the localization studies, however, and suggest that experiments are needed to validate the bioinformatic results, for example, fluorescence microscopy to validate the colocalization studies.

Another potential role of cytochrome b5 in giant viruses is as an antioxidant. Viral replication can stress the host cell, leading to reactive oxygen species (ROS) accumulation^30^ Studies have shown that overexpression of cytochrome b5 in mammalian cells reduces oxidative stress^31^ implying that cytochrome b5 in viruses might similarly safeguard the viral replication apparatus from ROS-induced damage. Nevertheless, the roles of cytochrome b5 in giant viruses likely span beyond a single function. Given its multifaceted nature, comprehensive molecular and biochemical studies are needed to decode specific contributions of cytochrome b5 within giant viruses, underscoring the need for further investigative work to clarify its exact function and role.

In a study of another gene family, the cytochrome P450s, genetically unrelated to the cytochrome b5s, we found it impossible to conclude that the P450 genes in the giant viruses were acquired from any known host^20^. However, some other viruses have P450s that appear to have been acquired from a known eukaryote host. Thus, hokovirus CYP5724A1 and *Ranid herpesvirus 3* CYP5723A1 share approximately 30% sequence identity to lipid and steroid metabolizing P450s present in their hosts, and the P450 102L1 we reported in the mycophage *Mycobacterium* phage Adler was acquired from the *Mycobacterium abscessus* host^20^. A similar picture appears with the cytochrome b5 genes. The cytochrome b5s found in the *Ostreococcus* phages do bear significant similarities to the cytochrome b5s in their algal hosts, while the cytochrome b5 genes in other viruses in the *Nucleocytoviricota* do not appear to have been derived from any known hosts. It would be informative to determine whether there is similarity to cytochrome b5 in any new hosts identified. Barring that, the giant virus cytochrome b5 genes do not appear to have originated through gene transfer from a contemporary host, but rather, as appears to be the case for some other *Megaviricetes* genes, the cytochrome b5 genes may have been transferred from ancient hosts no longer extant.

The major distinction in the presence of cytochrome P450 genes and cytochrome b5 genes among the giant viruses either indicates that these genes were acquired separately, or that if they were acquired together, that the cytochrome b5s were lost from the GC-rich clades. Alternatively, if the cytochrome b5 genes are de novo creations of the giant viruses, then that process would have been restricted, or the genes may have been lost along the way from the GC-rich clades. For both cytochrome b5 and cytochrome P450 there is an absolute requirement for heme cofactor, intimating that both viral gene families were transferred from a cellular host as no complete heme biosynthetic enzyme machinery has been found to be encoded in a giant virus genome to-date. Alternatively, viruses in the *Megaviricetes* may originally have possessed heme biosynthetic machinery but then lost it as it became possible to dependent on a host pathway for heme supply. In either case, the presence of genes for cytochromes b5 in the giant viruses adds to the growing number of genes in the giant viruses that are not expected in any virus, informing the discussion of the origin of the giant viruses and the genes they contain. In particular this study raises adds to questions about the origin of genes for heme proteins and their role in the giant viruses.

## Methods

### Viral cytochrome b5 sequence datasets

The cytochrome b5 domain is readily identifiable bioinformatically. Candidate viral cytochrome b5 sequences were retrieved from the NCBI sequence databases (online version as of December 15, 2024). Sequences homologous to mimivirus cytochrome b5 were identified in the environmental metagenomic database (env_nr) and the non-redundant database (nr).

The Genbank accession numbers for viruses here are given in Supplemental Table 1). Inferred protein sequences were aligned to the PfAM b5-domain hidden Markov model (PF00173) using ClustalOmega^32^, and scored with T-Coffee^33^. Alignments were visualized in BioEdit^34^. Virus names are those in the original reports or described in Genbank. Pairwise amino acid identities reported in the text are based on the length of the shortest protein in the alignment. Identity values used in the tables are based on multiple sequence alignments and are usually not more than 1% different from stated values.

### Phylogenetic analyses of viral cytochrome b5

Phylogenetic trees of cytochrome b5 were inferred using maximum likelihood (ML) approaches (RAxML-ng v1.2.2) with the VT + I + G4 model of amino acid substitution as selected by ModelTest^35^. Twenty replicate ML searches were conducted, and the best-scoring tree was subjected to rapid bootstrapping in RAxML to assess bifurcation support using the autoMRE bootstopping criteria, which produced 1000 bootstrap replicates. Virus phylogenies were adapted from the concatenated seven conserved gene set identified by Aylward et al (2021) by extensively pruning the published phylogeny^20^. Figures were constructed using iToL^36^.

### Modelling and structural analysis

Homology modelling of viral b5 proteins was performed using Modeller (v9.16)^37^. 100 independent models were produced based on rat, housefly, and *Ostreococcus* virus cytochrome b5 structures (PDB codes 4B8N,1EUE, 3MUS, 2IBJ). The model with the highest DOPE-HR (high resolution discrete optimized potential energy) score was selected and visualized in Pymol. Further structural analysis was performed using Thesesus^38^.

Subcellular colocalization of viral cytochrome b5 proteins was predicted using the DeepLoc 1.0 and DeepLoc 2.0 algorithms^39,40^ These algorithms differ slightly. By using both versions, we ensured that any observed differences were not due to algorithmic changes but instead gave meaningful biological insights. The significance of the predictions was determined based on the likelihood values provided by the DeepLoc algorithms. These values range from 0 to 1, where 0 indicates no likelihood of colocalization and 1 indicates absolute certainty of colocalization. Higher likelihood values suggest a stronger probability of colocalization with a given compartment.

### Mimivirus cytochrome b5 transcriptomic analysis and protein characterisation

The mimivirus cytochrome b5 gene (MIMI_L628) was synthesised by Eurofins MWG Operon and gene integrity confirmed by DNA sequencing. A unique *Nde*I restriction site was generated prior to the ATG start codon and a *Hin*dIII site following the TGA stop codon to facilitate cloning into the *Escherichia coli* expression plasmid, pET17b. Additionally, a polyhistidine tag, to allow purification of the expressed protein by Ni^2+^-NTA affinity chromatography, was engineered into the C-terminus of the protein. MIMI L628 was expressed and purified using similar conditions described for *Ostreococcus* virus cytochrome b5^19^. Cytochrome b5 content was quantified from the reduced versus oxidized difference spectrum of purified protein using a differential absorption coefficient Δ_424 – 409_ of 185 mM^−1^ cm^−1^^41^.

## Supplementary information

Supplemental Material_revised

## Data Availability

Data will be made available on request.
