# Supplementary material for: Cytochrome b5 occurrence in giant and other viruses belonging to the phylum Nucleocytoviricota
Source: Npj Viruses. 2025 Feb 11;3:8. doi: 10.1038/s44298-025-00091-3 (PMC11814380; doi:10.1038/s44298-025-00091-3)
Supplement: Supplementary file 1 — Supplemental Material_revised [file 44298_2025_91_MOESM1_ESM.pdf]

Supplemental Material for:

Cytochromes b5 Occurrence in Giant and Other Viruses Belonging to the Phylum *Nucleocytoviricota*

David C. Lamb[1], Jared V. Goldstone[2], Djamal Brahim Belhaouari[3], Julien Andréani[4,5], Ayesha Farooqi[1], Michael J. Allen[6], Steven L. Kelly[1], Bernard La Scola[4,5], and John J. Stegeman [2,*]

Supplemental Table 1. List of Genbank Accession numbers for cytochrome b5 from viruses discussed.

Supplemental Table 2. Details for intracellular localization for virus cytochrome b5 proteins.

Supplemental Figure 1. Maximum likelihood phylogenetic analysis of cytochrome b5-domain containing proteins. General functional classes are noted. Giant virus cytochrome b5 proteins are highlighted in red. 994 sequences were aligned with Clustal omega and analyzed with RAxML-ng (1.2.1)

Supplemental Figure 2. Synteny analysis of cytochrome b5 loci in *Ostreococcus* viruses. Loci and surrounding genes for cytochrome b5 genes are marked in strains of the *Ostreococcus* viruses. Note especially the prevalence of the Fe(II)-2-OGD, i.e., Fe(II)- and 2-oxoglutarate dependent dioxygenases.

Supplemental Table 1

Accession number	Order	Family	Species or other name	Name
ECG46315.1			MAG GOS_5664724	MAG: GOS_5664724 partial marine metagenome
ECC71381.1			MAG GOS_6325992	MAG: GOS_6325992 partial marine metagenome
ECU04489.1			MAG GOS_3560013	MAG: GOS_3560013 partial marine metagenome
EBL02824.1			MAG GOS_8635627	MAG: GOS_8635627 marine metagenome
ECB72846.1			MAG GOS_3236397	MAG: GOS_3236397 partial marine metagenome
EBG74059.1			MAG GOS_9382221	MAG: GOS_9382221 marine metagenome
QHU33303.1			MAG GVMAG-S-1014582-52	MAG: hypothetical protein viral metagenome
YP_010842063.1	Imitervirales	Mimiviridae	*Cotonvirus japonicus*	putative cytochrome B5-like Cotonvirus japonicus
YP_010777544.1	Imitervirales	Mimiviridae	*Mimivirus bradfordmassiliense*	cyt-b5 Acanthamoeba polyphaga mimivirus
AHJ40257.2	Imitervirales	Mimiviridae	*Mimivirus bradfordmassiliense* Samba	Cytochrome b5-like protein Samba virus
ALR84216.1	Imitervirales	Mimiviridae	*Mimivirus bradfordmassiliense* Niemeyer	Cytochrome b5-like protein Niemeyer virus
BAV62730.1	Imitervirales	Mimiviridae	*Mimivirus bradfordmassiliense* shirakome	cytochrome b5-like protein mimivirus shirakomae
BAV61744.1	Imitervirales	Mimiviridae	*Mimivirus bradfordmassiliense* kasai	cytochrome b5-like protein mimivirus kasaii
AHA45216.1	Imitervirales	Mimiviridae	*Mimivirus bradfordmassiliense* hirudovirus	cyt-b5 Hirudovirus strain Sangsue
AEQ60829.1	Imitervirales	Mimiviridae	*Mimivirus bradfordmassiliense* A castellani mamvirus	Cyt-b5 Acanthamoeba castellanii mamavirus
QBK86278.1	Imitervirales	Mimiviridae	Marseillevirus LCMAC102	MAG: Marseillevirus LCMAC102
AKI79407.1	Imitervirales	Mimiviridae	*Mimivirus bradfordmassiliense* strain	cyt-b5 Acanthamoeba polyphaga mimivirus
ANB50850.1	Imitervirales	Mimiviridae	*Megavirus powaiense*	cyt-b5 Powai lake megavirus
AFX92855.1	Imitervirales	Mimiviridae	*Megavirus chilense* courdo11	cyt-b5 Megavirus courdo11
AUV58705.1	Imitervirales	Mimiviridae	Megavirus chilense Bandra	Cytochrome B5 Bandra megavirus
AVL94070.1	Imitervirales	Mimiviridae	*Megavirus chilense* strain	Cytochrome b5-like binding domain Megavirus
AGD92703.1	Imitervirales	Mimiviridae	*Megavirus chilense* Iba	Cytochrome B5-like protein Megavirus lba
YP_004894807.1	Imitervirales	Mimiviridae	*Megavirus chilense*	cyt-b5 Megavirus chiliensis
YP_010788598.1	Imitervirales	Mimiviridae	*Megavirus baoshan*	cytochrome B5-like protein Megavirus baoshan
AKI80364.1	Imitervirales	Mimiviridae	*Mimivirus lagoaense*	cyt-b5 Kroon mimivirus
AGF85059.1	Imitervirales	Mimiviridae	*Moumouvirus goulettemassiliense*	cyt-b5 Moumouvirus goulette
AQN68565.1	Imitervirales	Mimiviridae	*Moumouvirus moumou* Saudi	cyt-b5 Saudi moumouvirus
YP_007354627.1	Imitervirales	Mimiviridae	*Moumouvirus moumou*	cytochrome b5-like Acanthamoeba polyphaga moumouvirus
BFL61643.1	Imitervirales	Mimiviridae	*Moumouvirus lavasanguinem*	cytochrome b5-like Moumouvirus lavasanguinem
AVL95051.1	Imitervirales	Mimiviridae	*Moumouvirus australiensis* castellanii 1	cyt-b5 Moumouvirus australiensis
YP_010789984.1	Imitervirales	Mimiviridae	*Moumouvirus australiensis* castellanii 2	B5-like protein Moumouvirus australiensis

Supplemental Table 1 (cont)

Accession number	Order	Family	Species or other name	Name
QGR54182.1	Imitervirales	Mimiviridae	*Moumouvirus maliensis*	cyt-b5 Moumouvirus maliensis
QID06377.1	Imitervirales	Mimiviridae	*Moumouvirus* strain Borley	Cytochrome b5-like protein Borely moumouvirus
QHT01011.1	Imitervirales	Mimiviridae	MAG GVMAG-M-3300020192-26	MAG: hypothetical protein viral metagenome
AYV84960.1	Imitervirales	Mimiviridae	MAG Satyrvirus1_46	cyt-b5 Satyrvirus sp.
YP_010780041.1	Imitervirales	Mimiviridae	*Tupanvirus altamarinense*	cytochrome b5-like Tupanvirus deep ocean
YP_010781319.1	Imitervirales	Mimiviridae	*Tupanvirus salinum*	cytochrome b5-like Tupanvirus soda lake
ARF11152.1	Imitervirales	Mimiviridae	MAG Klosneuvirus1_9	hypothetical protein Klosneuvirus_1_9 Klosneuvirus KNV1
YP_010802999.1	Imitervirales	Mimiviridae	*Fadolivirus algeromassiliense*	cytochrome b5-like protein Fadolivirus algeromassiliense
YP_004063634.1	Algavirales	Phycodnaviridae	*Ostreococcus tauri* virus OtV2	Ostreococcus tauri virus OtV2
AET84650.1	Algavirales	Phycodnaviridae	*Ostreococcus lucimarinus* OlV4	Ostreococcus lucimarinus OlV4
QIG59825.1	Algavirales	Phycodnaviridae	*Prasinovirus* spp.	Dishui lake phycodnavirus 3
ECF09823.1			MAG GOS_4079050	MAG: GOS_4079050 marine metagenome
ECC19277.1			MAGGOS_4848604	MAG:GOS_4848604 marine metagenome
EBX20320.1			MAG GOS_6624691	MAG: GOS_6624691 partial marine metagenome
ECY61807.1			MAG GOS_2328651	MAG: GOS_2328651 partial marine metagenome
ECV95134.1			MAG GOS_2806517	MAG: GOS_2806517 marine metagenome
AYV75195.1	Imitervirales	Mimiviridae	Terrestrivirus	MAG: putative cytochrome b5 Terrestrivirus sp.
AYV80661.1	Imitervirales	Mimiviridae	Harfovirus	MAG: putative Cytochrome b5 Harvfovirus sp.
AYV84005.1	Imitervirales	Mimiviridae	Hyperionvirus	MAG: hypothetical Hyperionvirus15_43 Hyperionvirus sp.

Supplemental Table 2.

Taxon	Species	Protein_ID	Localizations	Signals	Cytoplasm	Nucleus	Extracellular	Cell membrane	Mitochondrion	Plastid	Endoplasmic reticulum	Lysosome/Vacuole	Golgi apparatus	Peroxisome
Mimiviruses	Mimivirus	Q5UR80.1	ER	Signal peptide	0.287	0.261	0.136	0.398	0.374	0.047	0.832	0.109	0.212	0.382
	Mamavirus	AEQ60829.1	ER	Signal peptide	0.284	0.266	0.115	0.373	0.379	0.044	0.838	0.105	0.202	0.400
	Oyster virus	AKI79407.1	Cytoplasm	Peroxisomal targeting	0.794	0.425	0.028	0.154	0.261	0.011	0.357	0.046	0.079	0.259
	Kroon virus	AKI80364.1	Cytoplasm	Peroxisomal targeting	0.798	0.431	0.027	0.132	0.317	0.013	0.360	0.045	0.082	0.275
	Hirudovirus	AHA45216.1	Cytoplasm	Peroxisomal targeting	0.799	0.396	0.031	0.173	0.248	0.011	0.342	0.056	0.081	0.228
	Niemeyer virus	ALR84216.1	ER	Signal peptide	0.287	0.261	0.136	0.398	0.374	0.047	0.832	0.109	0.212	0.382
	Bombay virus	AMZ03071.1	ER	Signal peptide	0.287	0.261	0.136	0.398	0.374	0.047	0.832	0.109	0.212	0.382
	Shirakomae virus	BAV62730.1	Cytoplasm	Peroxisomal targeting	0.799	0.396	0.031	0.173	0.248	0.011	0.342	0.056	0.081	0.228
	Kasaii virus	BAV61744.1	Cytoplasm	Peroxisomal targeting	0.799	0.396	0.031	0.173	0.248	0.011	0.342	0.056	0.081	0.228
	Samba virus	AHJ40257.2	Cytoplasm	Peroxisomal targeting	0.799	0.396	0.031	0.173	0.248	0.011	0.342	0.056	0.081	0.228
Moumouviruses	Acanthamoeba polyphaga moumouvirus	AGC02191.1	ER	Signal peptide	0.319	0.289	0.126	0.226	0.248	0.024	0.864	0.162	0.218	0.427
	Moumouvirus monve	AEX62481.1	ER	Signal peptide	0.319	0.289	0.126	0.226	0.248	0.024	0.864	0.162	0.218	0.427
	Saudi moumouvirus	AQN68565.1	ER	Signal peptide	0.324	0.268	0.103	0.237	0.258	0.024	0.914	0.167	0.253	0.504
	Moumouvirus goulette	AGF85059.1	ER	Signal peptide	0.356	0.294	0.133	0.191	0.250	0.020	0.865	0.176	0.299	0.473
	Moumouvirus australiensis	AVL95051.1	Cytoplasm		0.896	0.427	0.052	0.107	0.116	0.039	0.320	0.122	0.264	0.018
	Moumouvirus maliensis	QGR54182.1	ER	Signal peptide	0.343	0.301	0.140	0.218	0.260	0.028	0.859	0.181	0.264	0.340
	Borely moumouvirus	QID06377.1	ER	Signal peptide	0.334	0.302	0.140	0.232	0.250	0.029	0.863	0.184	0.249	0.354
Megaviruses	Megavirus chiliensis	AEQ33147.1	ER	Signal peptide	0.442	0.321	0.266	0.342	0.291	0.031	0.814	0.141	0.277	0.197
	Mimivirus lba	AGD92703.1	Cytoplasm	Peroxisomal targeting	0.877	0.486	0.015	0.178	0.224	0.004	0.491	0.069	0.038	0.309
	Megavirus courdo11	AFX92855.1	Cytoplasm	Peroxisomal targeting	0.877	0.486	0.015	0.178	0.224	0.004	0.491	0.069	0.038	0.309
	Powai Lake Megavirus	ANB50850.1	ER	Signal peptide	0.455	0.301	0.266	0.326	0.279	0.024	0.797	0.145	0.267	0.128
	Bandra Megavirus	AUV58705.1	ER	Signal peptide	0.442	0.321	0.266	0.342	0.291	0.031	0.814	0.141	0.277	0.197
	Megavirus vitis	AVL94070.1	Cytoplasm	Peroxisomal targeting	0.877	0.486	0.015	0.178	0.224	0.004	0.491	0.069	0.038	0.309
	Megavirus baoshan	AZL89108.1	ER	Signal peptide	0.448	0.309	0.271	0.354	0.289	0.028	0.809	0.151	0.264	0.191
Tupanviruses	Tupanvirus (Deep Ocean 3,000 m)	QKU33443.1	ER	Signal peptide|Transmembrane domain	0.253	0.241	0.066	0.177	0.147	0.013	0.913	0.412	0.269	0.036
	Tupanvirus (Soda Lake)	QKU34676.1	ER	Signal peptide|Transmembrane domain	0.266	0.269	0.060	0.172	0.306	0.027	0.890	0.273	0.257	0.058
Satyrvirus	Satyrvirus sp. (metagenome)	AYV84960.1	Cytoplasm	Peroxisomal targeting	0.858	0.403	0.046	0.096	0.330	0.018	0.416	0.109	0.097	0.113
Marseillevirus	LCMAC102 (metagenome)	QBK86278.1	Cytoplasm		0.785	0.422	0.218	0.140	0.159	0.039	0.356	0.093	0.235	0.040
Ostreococcus lucimarinus virus	Olv1	ADQ91589.1	Cytoplasm	Peroxisomal targeting	0.766	0.226	0.124	0.294	0.415	0.069	0.463	0.113	0.118	0.097
	Olv2	YP_009172721.1	Cytoplasm	Peroxisomal targeting	0.767	0.274	0.020	0.282	0.385	0.036	0.583	0.145	0.169	0.090
	Olv3	AFK66042.1	Cytoplasm	Peroxisomal targeting	0.753	0.288	0.020	0.305	0.374	0.036	0.563	0.157	0.172	0.089
	Olv4	AET84663.1	Cytoplasm		0.777	0.271	0.059	0.265	0.338	0.046	0.443	0.168	0.127	0.045
	Olv5	AGH31115.1	Cytoplasm		0.774	0.298	0.050	0.254	0.320	0.048	0.447	0.153	0.126	0.042
	Olv6	AFK65792.1	Cytoplasm		0.774	0.298	0.050	0.254	0.320	0.048	0.447	0.153	0.126	0.042
	Olv7	ALI95841.1	Cytoplasm		0.773	0.229	0.140	0.292	0.408	0.065	0.449	0.108	0.114	0.083

Supplemental Table 2 (cont).

Taxon	Species	Protein_ID	Localizations	Signals	Cytoplasm	Nucleus	Extracellular	Cell membrane	Mitochondrion	Plastid	Endoplasmic reticulum	Lysosome/Vacuole	Golgi apparatus	Peroxisome
Ostreococcus tauri virus	OtV2	YP_004063634.1	Cytoplasm		0.774	0.298	0.050	0.254	0.320	0.048	0.447	0.153	0.126	0.042
Homo sapiens		NP_683725.1	ER	Transmembrane domain	0.103	0.080	0.021	0.343	0.554	0.129	0.854	0.446	0.441	0.551
Acanthamoeba castellanii	Neff	ELR24118.1	ER	Signal peptide|Transmembrane domain	0.089	0.118	0.022	0.173	0.550	0.111	0.874	0.213	0.263	0.165

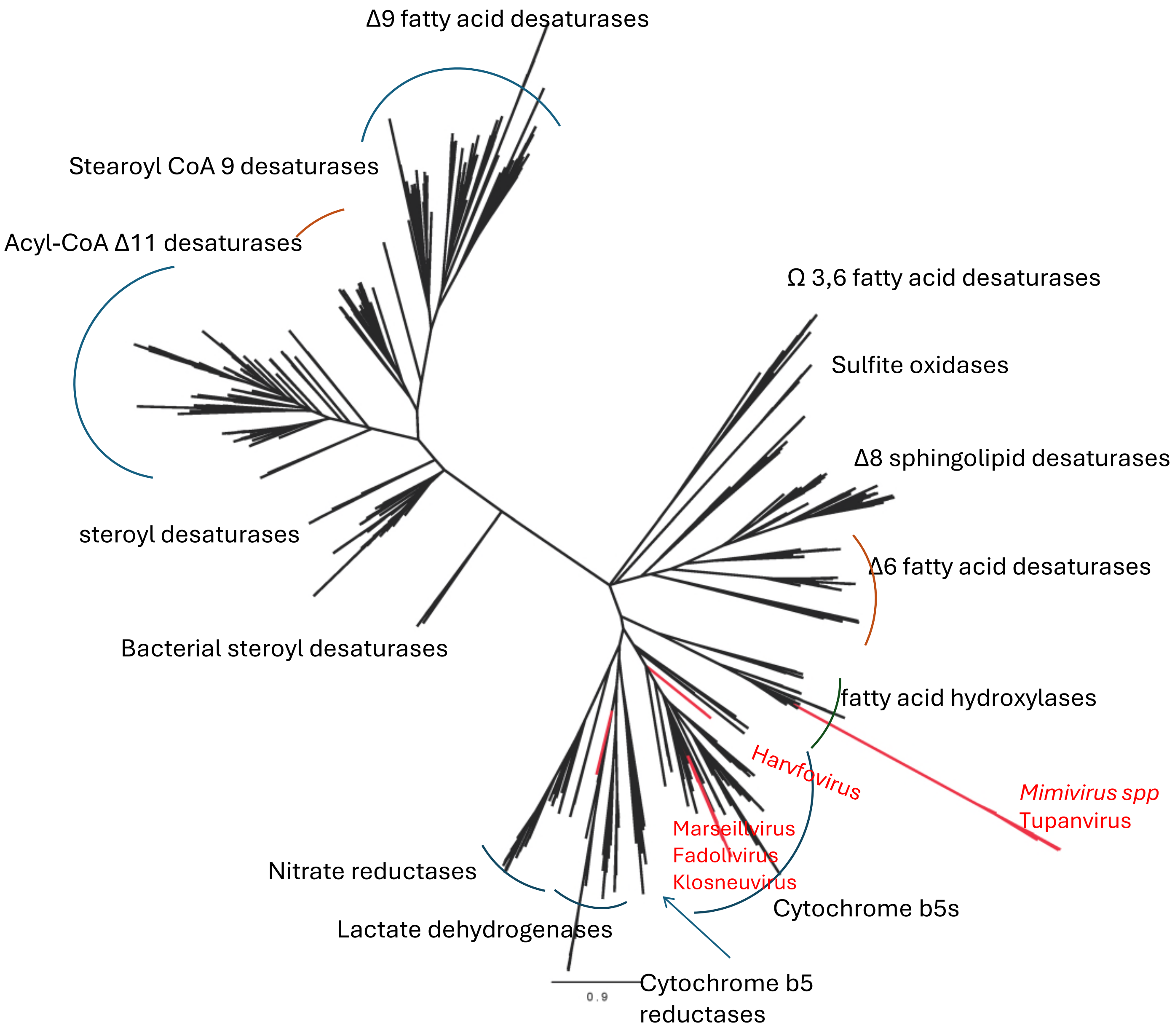

Supplemental Figure 1. **Maximum likelihood phylogenetic analysis of cytochrome b5-domain containing proteins.** General functional classes are noted. Giant virus cytochrome b5 proteins are highlighted in red. 994 sequences were aligned with Clustal omega and analyzed with RAxML-ng (1.2.1)

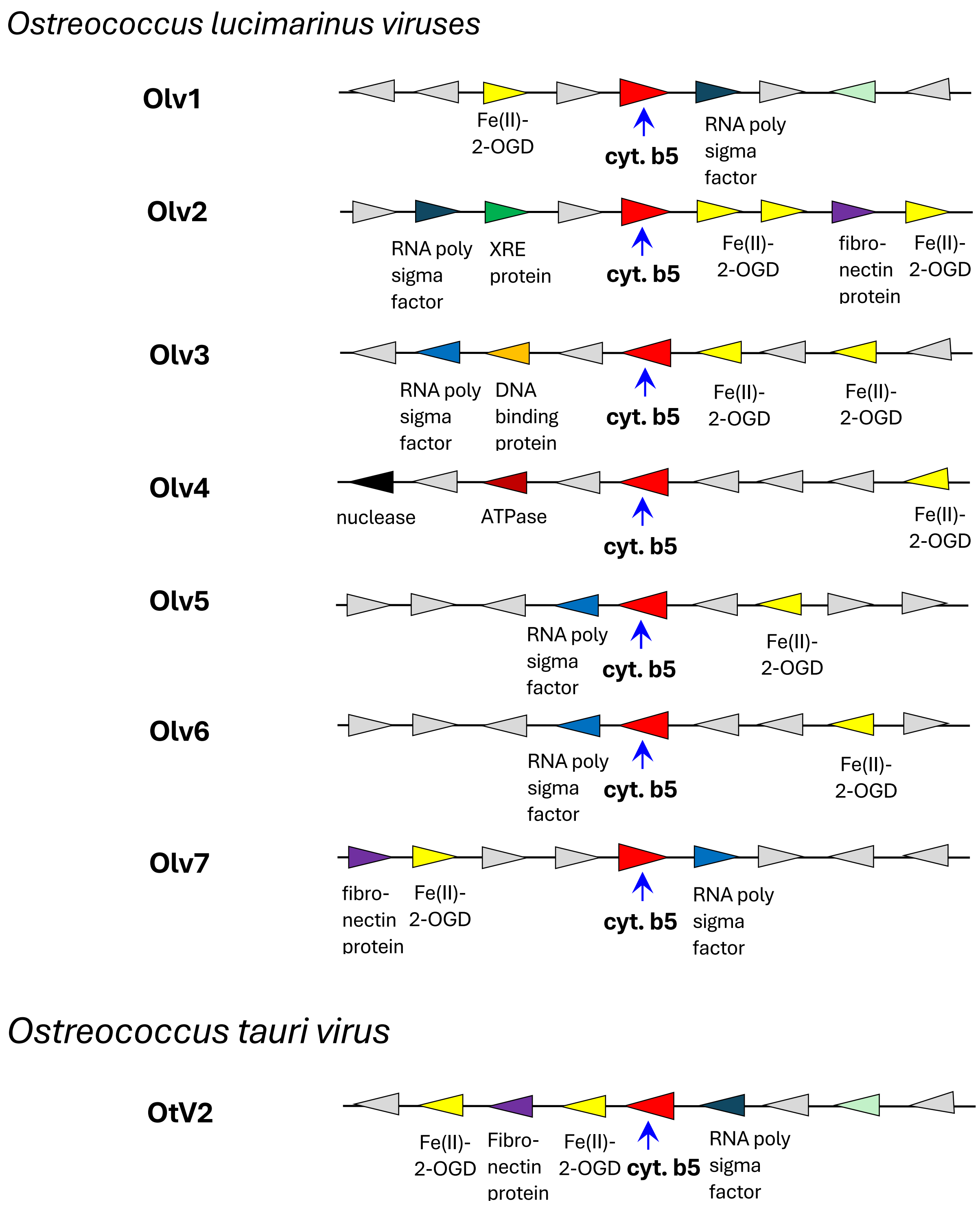

Supplemental Figure 2. Synteny analysis of cytochrome b5 loci in *Ostreococcus* viruses. Loci and surrounding genes for cytochrome b5 genes in strains of the *Ostreococcus* viruses. Note especially the prevalence of the Fe(II)-2-OGD, i.e., Fe(II)- and 2-oxoglutarate dependent dioxygenases.
